# Psychedelics and the quantum brain: a falsifiable hypothesis on Posner molecules and spin-dependent pharmacology

**DOI:** 10.3389/fphar.2026.1777613

**Published:** 2026-03-31

**Authors:** Joseph Geraci, Erik Viirre, Bessi Qorri, Luca Pani

**Affiliations:** 1 NetraMark Corp., Toronto, ON, Canada; 2 Department of Pathology and Molecular Sciences, Queen’s University, Kingston, ON, Canada; 3 Tandem Centre for Pharmacogenetics, Molecular Brain Science Department, Centre for Addiction and Mental Health, Toronto, ON, Canada; 4 Arthur C. Clarke Centre for Human Imagination, School of Physical Sciences, University of California, San Diego, CA, United States; 5 Department of Neurosciences, University of California, San Diego, CA, United States; 6 Department of Psychiatry and Behavioral Sciences, Leonard M. Miller School of Medicine, University of Miami, Coral Gables, FL, United States; 7 Department of Biomedical, Metabolic, and Neural Sciences, University of Modena and Reggio Emilia, Modena, Italy

**Keywords:** psychedelics, 5-HT2A receptor, Posner molecules, nuclear spin, quantum biology, precision psychiatry, clinical trials, treatment response

## Abstract

Classical serotonergic psychedelics (e.g., LSD, psilocybin, DMT) alter perception and neuroplasticity primarily via 5-HT2A receptor activation and downstream Ca^2+^-dependent signaling cascades. Here we propose a speculative yet falsifiable pharmacological hypothesis that these drug-induced biochemical cascades might interface with quantum-mechanical processes in the brain. We focus on nuclear spin dynamics in phosphate-containing biomolecules–calcium phosphate nanoclusters known as “Posner molecules” (Ca_9_(PO_4_)_6_) – as a candidate substrate for quantum coherence and entanglement in neural tissue. We distinguish the metaphorical “classical” analogies in psychedelic neuroscience from a literal quantum-level mechanism involving nuclear spin coherence and entanglement. The central hypothesis is that intense 5-HT2A-driven neural activity and Ca^2+^ flux during psychedelic exposure foster conditions under which ^31^P nuclear spins in phosphate groups may become entangled and shielded from decoherence within Posner molecules and subsequently influence neuronal signaling when these clusters dissolve and release bursts of Ca^2+^ in different neuronal compartments. Building on Fisher’s Posner model of quantum cognition, we reframe Posner molecules as a potential quantum-coherence nexus in psychedelic action, de-emphasizing earlier microtubule-centric models and explore how such quantum effects, if they exist, might influence pharmacological outcomes. We outline translational implications of this hypothesis, including potential insights into inter-individual variability in treatment response and novel experimental paradigms for psychiatry. To ensure falsifiability, we propose concrete experimental directions in the short term (isotopically modified psychedelics and xenon environments), medium term (advanced quantum sensors such as nitrogen-vacancy magnetometry and ultrafast spectroscopy), and long term (entangled ligand studies or quantum neuroimaging modalities). While speculative, this interdisciplinary framework generates specific, disprovable predictions. Confirming or refuting the role of quantum-mechanical phenomena in psychedelic neuropharmacology would profoundly impact our understanding of mind-brain relationships and encourage high-reward innovation in psychiatric treatment and brain-targeted drug design.

## 1 Introduction

Classic psychedelic drugs such as lysergic acid diethylamide (LSD), psilocybin/psilocin, and N,N-dimethyltryptamine (DMT) are potent 5-HT2A serotonin receptor subtype agonists that produce profound alterations in perception, affect, and self-experience (Nichols, 2016; Nichols et al., 2017). Activation of 5-HT2A receptors on layer V pyramidal neurons triggers G_q/11_-coupled phosphoinositide signaling, phospholipase C activation, inositol 1,4,5-triphosphate (IP_3_) production, and intracellular Ca^2+^ release, ultimately increasing cortical excitability and glutamatergic outflow (Nichols, 2016; Kwan et al., 2022).

Neuroimaging studies show that psychedelics acutely disrupt canonical large-scale networks such as the default mode network (DMN) and increase global functional connectivity, leading to a more integrated and less modular brain state (Nichols, 2016; Muthukumaraswamy et al., 2013; Tagliazucchi et al., 2014; Carhart-Harris et al., 2014). These observations underpin theoretical frameworks such as the “entropic brain” hypothesis and the REBUS (“Relaxed Beliefs Under Psychedelics”) model, which describe the psychedelic state as a high-entropy, more flexible mode of brain organization that temporarily relaxes top-down priors and permits cognitive and emotional reorganization (Carhart-Harris et al., 2014; Carhart-Harris and Friston, 2019). These classical frameworks employ analogies from thermodynamics and information theory (entropy, energy landscapes, criticality) to describe emergent neural dynamics.

While these models have proven useful, they stop short of positing physical quantum processes in the brain. They are essentially high-level analogies: increased entropy in neural activity is a metaphor for diversifying connectivity patterns, not a statement about thermodynamic entropy at the molecular scale. In contrast, here we explore the possibility that there may be a more literal quantum-mechanical aspect to how psychedelics act–one involving properties like spin, coherence, and entanglement of subatomic particles. This aligns with growing recognition in quantum biology that non-trivial quantum effects such as spin-dependent chemical reactions can occur in warm, wet biological environments. Well-established examples include the radical-pair mechanism in avian magnetoreception, where birds sense Earth’s magnetic field via quantum spin dynamics in cryptochrome proteins (Hore and Mouritsen, 2016; Mouritsen, 2018). Notably, even general anesthetic action has been speculatively linked to quantum spin effects such as xenon anesthesia and lithium’s mood-stabilizing action (Zadeh-Haghighi and Simon, 2022; Zadeh-Haghighi and Simon, 2021). These precedents motivate the question: Could certain neuropharmacological effects of psychedelics likewise involve quantum degrees of freedom?

Historically, quantum brain theories have focused on very different substrates, most prominently the microtubule hypothesis of consciousness (Orch-OR theory). Orch-OR posits that coherent quantum states inside neuronal microtubule proteins underlie conscious moments, with wavefunction collapse (“objective reduction”) orchestrated by biology (Hamer et al., 2014). While provocative, the microtubule theory remains controversial and has not been empirically confirmed, with arguments that decoherence at brain temperature would destroy such quantum states almost instantly. In light of these objections, we do not center microtubules here and instead focus on nuclear spins in smaller inorganic clusters.

In this work, we adopt a new perspective centered on the nuclear spins of certain atoms as candidate qubits in the brain. This shift is inspired by proposals from Fisher and colleagues that phosphorus nuclear spins in biomolecules could retain coherence for biologically relevant timescales (Fisher, 2015). This theoretical work identified the phosphate ion (PO_4_^3-^) as a potential “neural qubit” and calcium phosphate clusters, the Posner molecules, Ca_9_(PO_4_)_6_ as possible nano-cavities that protect entangled spin states. The Posner molecule hypothesis suggests a mechanism for quantum processing in neurons that is fundamentally different from Orch-OR, involving chemical reactions and nuclear spin entanglement rather than tubulin protein states.

It is important to note that experimental work has already begun to test concrete predictions relating calcium availability and calcium isotope dependence to neural processing. Chen et al. tested a Posner-based consciousness proposal that predicted sensitivity to calcium concentration and calcium isotope and reported no isotope dependence under their anesthesia paradigm (Chen et al., 2020). These negative results constrain that particular mechanism rather than ruling out the broader possibility that quantum-sensitive chemical processes could, under more restricted conditions, influence neural dynamics.

Our framework builds explicitly on these constraints. We focus on physiological feasibility bounds for cluster formation and lifetime, propose transient correlated microevents rather than persistent coherent states, and embed these within classical amplification and network-dynamics constraints (including latency-refractory constraints). In this way, Chen et al. helps define boundary conditions and motivates sharper experiments, without by itself falsifying the more constrained experimentally differentiated hypotheses advanced here.

Crucially, this hypothesis connects to pharmacology via calcium and phosphate metabolism. Posner molecules consist of nine Ca^2+^ and six phosphate ions formed *in vitro* and thought to be stabilized in bodily fluids, including perhaps the brain’s extracellular or intracellular environments. Fisher proposed that when an enzyme hydrolyzes pyrophosphate (PP_i_→2P_i_), the two resulting phosphate ions may emerge in an entangled nuclear spin state (Fisher, 2015). If each entangled phosphate enters a Posner molecule, the entanglement can be preserved as long as the Posner cluster remains intact. Neurons could hypothetically take up Posner molecules (e.g., via endocytosis or during neurotransmitter vesicle cycling) and transport them to presynaptic terminals. When two Posner molecules carrying entangled spins eventually come together and bind, a chemical reaction (“Posner bimolecular reaction”) may cause them to dissolve, releasing a burst of Ca^2+^ ions in two locations simultaneously. This release of Ca^2+^ could trigger synchronous neurotransmitter release or firing in the two affected neurons, creating a non-local correlation in neural activity. In essence, the Posner molecule acts as a quantum-coherent neuromodulator: it can distribute entanglement and then, upon disintegration, yield coordinated calcium signals that influence synaptic events in multiple neurons at once.

In this paper, we explore the possibility that classic psychedelic pharmacology may interact with such quantum-level processes. Specifically, we examine whether 5-HT2A-driven Ca^2+^ signaling and phosphate metabolism could engage nuclear-spin-based mechanisms involving Posner molecules as originally proposed in Fisher’s quantum cognition model. We aim to recast Posner molecules as a potential quantum-coherence substrate relevant to psychedelic action and distinguish metaphorical quantum-like analogies from a literal quantum-mechanical mechanism. We therefore propose a constrained, falsifiable hypothesis that links 5-HT2A-driven Ca^2+^/phosphate flux to transient Posner-like nanophases and spin-sensitive readout via correlated Ca^2+^ microevents. We pre-specify gating assays, discriminative spin perturbations, and circuit-level signatures that can decisively falsify the mechanism. Our intention is not to displace classical models of psychedelic action, but to formulate an additional, clearly testable layer that may or may not prove necessary to explain certain features of psychedelic experience and therapeutic response. By reframing psychedelic mechanisms to include such quantum effects, we hope to inspire new research bridging neuropharmacology, quantum chemistry, and experimental neuroscience.

## 2 Classical vs. quantum perspectives in psychedelic neuroscience

Classic psychedelics act as high-affinity 5-HT2A partial agonists that modulate cortical microcircuits, enhance glutamatergic transmission, and engage neuroplasticity-related pathways, including TrkB/BDNF and mTOR signaling (Nichols, 2016; Kwan et al., 2022; Ly et al., 2018; de Vos et al., 2021; Hesselgrave et al., 2021). At the systems level, fMRI, MEG, and EEG studies consistently demonstrate reduced integrity of the DMN and other high-order networks together with increased global functional connectivity and a richer repertoire of brain-state transitions (Muthukumaraswamy et al., 2013; Tagliazucchi et al., 2014). These observations motivated the entropic brain hypothesis, which proposes that psychedelic states correspond to higher entropy–greater dynamical diversity and unpredictability in key brain networks–correlating with the fluidity of conscious contents (Carhart-Harris et al., 2014). Such frameworks borrow concepts from physics but apply them metaphorically, at the level of emergent neural dynamics rather than molecular thermodynamics.

In contrast, a quantum-mechanical perspective asks whether microscopic quantum states (wavefunctions of particles) play an active role in psychedelic neurobiology. Relevant quantum features include coherence, the persistence of phase relationships between quantum states, and entanglement, the non-classical correlation of measurement outcomes between spatially separated particles. In biological environments, such effects are most plausibly associated with nuclear spins or certain electron spins rather than collective excitations of large proteins. Nuclear spins in ubiquitous biomolecules (e.g., ^31^P in phosphate groups, ^1^H in water or organic molecules) are attractive candidates because they couple weakly to environmental noise and may exhibit longer coherence times than electronic or vibrational states.

Quantum biology provides precedents for functional spin-dependent reactions, most notably the radical-pair mechanism of avian magnetoreception in non-neural tissues (Hore and Mouritsen, 2016). In cryptochrome proteins, photo-excited electron pairs become spin-entangled, and singlet-triplet interconversion driven by weak magnetic fields enables directional sensing (Hore and Mouritsen, 2016; Zadeh-Haghighi and Simon, 2022). This mechanism leverages internal nuclear spins to create magnetic anisotropies that influence spin dynamics, demonstrating that biological systems can exploit quantum spin effects.

Analogously, we ask whether nuclear-spin-dependent processes in the brain could modulate neuronal signaling. Phosphorus is a unique candidate in this respect. Fisher’s analysis suggested that freely diffusing phosphate ions may retain ^31^P spin coherence for tens to hundreds of seconds under low-noise conditions and identified the calcium phosphate cluster Ca_9_(PO_4_)_6_ (the Posner molecule) as a structure capable of shielding embedded phosphate spins from decoherence (Fisher, 2015). Unlike microtubule-based proposals, Posner molecules are small inorganic clusters whose symmetry and NMR properties could, in principle, sustain coherence at physiological temperatures. Their spin dynamics would manifest through chemical association and dissociation events with direct consequences for Ca^2+^ release, a central signal in neuronal physiology.

Classical models therefore describe the brain as a high-dimensional adaptive system governed by statistical dynamics, whereas a quantum account focuses on specific molecular-scale quantum states that may influence those dynamics. These perspectives are not mutually exclusive: if present, quantum events would feed into classical network behavior. The distinction is that the quantum framework introduces qualitatively new models of interaction, such as entanglement-mediated correlations in Ca^2+^ signaling, that lack classical counterparts. The following sections outline how such processes could arise via Posner molecules during psychedelic-induced physiological states and what implications they might hold.

## 3 Posner molecules as a quantum-coherence candidate in the brain

### 3.1 The Posner molecule hypothesis

The Posner molecule (Ca_9_(PO_4_)_6_) was proposed by Fisher as a chemically plausible host for quantum processing in the brain based on nuclear spins of phosphorus (^31^P) (Fisher, 2015). In this framework, phosphorus nuclei serve as neural qubits and the phosphate ion (PO_4_^3-^) is their primary carrier. Posner molecules are small calcium-phosphate nanoclusters that can be viewed as two Ca_3_(PO_4_)_2_ associated with three additional Ca^2+^ ions, producing an approximately 1 nm cluster with high symmetry (Swift et al., 2018).

A critical hypothesized step in the model is the enzymatic hydrolysis of pyrophosphate, PP_i_ → 2P_i_, which produces two phosphate ions whose ^31^P nuclear spins may be entangled in a singlet state. Each entangled phosphate can then be incorporated into a different Posner molecule, transferring entanglement into the overall nuclear spin states of the clusters (Fisher, 2015). Due to the symmetry and NMR properties of these clusters, the six ^31^P nuclei in a Posner molecule are predicted to be relatively protected from decoherence for timescales far longer than typical molecular environments would allow (Fisher, 2015; Yunger Halpern and Crosson, 2019).

Theoretical and computational studies using density functional theory and molecular dynamics have refined this picture and support the chemical plausibility of Ca_9_(PO_4_)_6_ clusters in simulated body fluid. These analyses indicate that the internal ^31^P environments could, in principle, sustain long-lived spin states (Swift et al., 2018; Yunger Halpern and Crosson, 2019). Although Posner molecules have not yet been demonstrated *in vivo* and are treated here as a testable substrate not an established entity, these results support the underlying idea that such structures could function as a quantum memory element.

Fisher also proposed that Posner molecules formed in extracellular fluid could be taken up into presynaptic terminals during vesicle cycling and transported within axons (Fisher, 2015; Weingarten et al., 2016). When two Posner molecules carrying entangled nuclear spins bind and dissolve, the resulting release of Ca^2+^ in two distant locations converts the nuclear spin entanglement into synchronized Ca^2+^ transients that may bias neurotransmitter release or spike timing (Fisher, 2015; Weingarten et al., 2016).

### 3.2 Chemical plausibility in neural tissue

For this mechanism to be relevant, Posner-like clusters must be chemically feasible in neural environments. Calcium and phosphate are abundant and tightly regulated in the central nervous system. Calcium serves as a core second messenger for neurotransmitter release, synaptic plasticity, gene transcription, and cell survival, while phosphate participates in ATP metabolism, nucleic acids, phospholipids, and a wide range of signaling pathways (Pikor et al., 2024; Kritmetapak and Kumar, 2019; Ghibelli et al., 2010). Intense neural activity produces local bursts of Ca^2+^ influx and elevated rates of ATP hydrolysis, which transiently increase P_i_ and PP_i_ levels. This combination can create conditions in restricted microdomains such as synapses, dendritic spines, and the mitochondrial matrix where calcium phosphate nanoclusters may nucleate.

By framing mitochondria as active “reactors” that concentrate Ca^2+^ and phosphate, rather than merely competitors for free cytosolic ions, the model accounts for the spatial and temporal scales required for nanocluster formation. These granules are natively integrated into neuronal calcium handling and the metabolic flux characteristic of high-intensity signaling states, including those induced by psychedelic exposure. The sequestered environment within the mitochondrial matrix also provides a degree of protection from cytosolic buffering and competition from Mg^2+^ or other chelators, favoring the metastability of structured calcium-phosphate phases like the Posner molecule (Delacour et al., 2025; Posner and Betts, 2002; Pivovarova and Andrews, 2010).

While the proposed mechanism involves non-classical spin dynamics, it is fundamentally grounded in the established constraints of neuronal calcium handling. We acknowledge seminal skeptical analyses emphasizing that many electronic and vibrational degrees of freedom decohere extremely rapidly in the brain’s warm, wet, and noisy environment (Tegmark, 2000). However, our model specifically focuses on nuclear spins within the high symmetry Ca_9_(PO_4_)_6_ cluster, which are hypothesized to be uniquely shielded from these environmental interactions.

The biological feasibility of this substrate is further supported by the existence of highly localized calcium signaling nanodomains, where restricted diffusion and rapid influx create transient zones of ionic supersaturation (Augustine et al., 2003; Neher, 1998). Under these conditions, the formation of inorganic calcium-phosphate phases is not merely speculative but a documented feature of neural physiology. Specifically, mitochondria function as dynamic ion reservoirs that precipitate amorphous calcium-phosphate granules as a core component of their buffering capacity, and recent high-resolution imaging suggests that primary cortical neurons can precipitate and even extrude large mitochondria-associated calcium-phosphate sheets (Pivovarova and Andrews, 2010; Anderson et al., 2026). By framing the Posner molecule as a structured, quantum-sensitive candidate within these endogenous Ca-P systems, we provide a classical comparator that aligns the hypothesis with measurable features of mitochondrial and synaptic biology.

Recent work on lithium isotopes provides a potential link between calcium phosphate nanophases and neuropsychiatric effects. Lithium isotopes have been shown to alter the size and distribution of mitochondrial amorphous calcium phosphate granules in brain tissue, indicating that nuclear spin can influence the behavior of these inorganic clusters (Delacour et al., 2025).

Although Posner molecules have not been directly detected in neural tissue, Ca_9_(PO_4_)_6_ clusters have been synthesized *in vitro* and are predicted to be metastable under near-physiological ionic strengths (Swift et al., 2018). These findings support the broader plausibility that ordered or partially ordered calcium phosphate structures could form in brain environments experiencing repeated high-frequency activity and metabolic flux. In this view, Posner molecules represent a specific structured subset of a more general family of calcium phosphate clusters already implicated in mitochondrial calcium handling and possibly in mood regulation through lithium isotope effects.

### 3.3 From nuclear entanglement to network-level effects

The conceptual appeal of the Posner hypothesis is its biochemical specificity and testability relative to earlier quantum brain proposals. Rather than invoking large protein assemblies, the Posner model centers on identifiable ionic species, defined nanoclusters, and explicit chemical reactions that can be examined with spectroscopy, isotope substitution, and structural methods (Hamer et al., 2014).

Mechanistically, enzymatic cleavage of PP_i_ generates entangled phosphate pairs whose ^31^P nuclear spins occupy a singlet state (Fisher, 2015). Each phosphate can enter a different Posner molecule, embedding entanglement into two separate clusters. These Posner molecules may then diffuse or be transported to distinct synaptic terminals within the same neuron or across neurons (Weingarten et al., 2016). When local biochemical conditions favor Posner binding and dissolution, the stored entanglement is converted into correlated Ca^2+^ release at the two sites (Fisher, 2015; Weingarten et al., 2016). Even modest correlations in calcium dynamics can modulate neurotransmitter release probability or spike synchrony. A key unresolved question is how a nuclear-spin state could plausibly modulate a chemical event that matters for synaptic physiology.

We hypothesize that the transduction of nuclear spin information into neuronal signaling is governed by symmetry-gated chemical kinetics at the level of individual Posner molecule dissolution events. The primary coupling mechanism is rooted in the high-symmetry S_6_ geometry of the Posner cluster, Ca_9_(PO_4_)_6_ (Fisher, 2015; Swift et al., 2018). In this configuration, the six phosphate groups occupy equivalent positions in a cage coordinated by nine calcium ions, a structure predicted to be relatively protected from environmental decoherence. Aqueous dissolution of this cluster would not be a purely stochastic thermal event but gated by the singlet-triplet configuration of the entangled ^31^P nuclear spin pairs within the cluster. A coherent singlet state preserves the structural symmetry required for the cluster’s metastability, suppressing premature dissolution, whereas decoherence into the triplet manifold breaks this symmetry and lowers the energetic barrier to hydration and ionic release (Fisher, 2015; Yunger Halpern and Crosson, 2019). Critically, when two entangled clusters share a common singlet state, their symmetry breaking, and therefore their dissolution, is correlated in time, even across spatial separation, effectively turning the nuclear spin state into a quantum “gate” for coordinated ionic release rather than simply a switch governing the rate of a single cluster (Fisher, 2015).

To scale above the brain’s thermal noise floor, the model utilizes a mesoscopic amplification step. Dissolution of a single Posner cluster releases a burst of ∼nine Ca^2+^ ions and six phosphate ions into the local microdomain (Fisher, 2015; Weingarten et al., 2016). This rapid release provides biochemical gain, converting a molecular-scale quantum event into a macroscopic Ca^2+^ transient capable of influencing high-sensitivity biological targets, such as the Ca^2+^-sensing machinery involved in neurotransmitter vesicle fusion. This amplification ensures that the quantum signal achieves a sufficient signal-to-noise ratio to impact synaptic events before being absorbed by the neuron’s standard buffering systems (Pikor et al., 2024; Vargas et al., 2023).

The final transduction into circuit-level effects occurs through a distributed excitability bias within local ionic microenvironments. Rather than a bulk “bath” effect, we hypothesize that the correlated release of Ca^2+^ from entangled Posner molecules across spatially separated synapses subtly tunes the membrane excitability and coincidence-detection windows within synaptic nanodomains or dendritic spine heads (Fisher, 2015; Augustine et al., 2003; Neher, 1998). This modulation is plausibly most pronounced when the cortical network operates near a critical point between ordered and disordered dynamics, a regime in which correlated subthreshold perturbations are not averaged away but instead produce measurable shifts in population-level electrical activity (Carhart-Harris et al., 2014; Carhart-Harris and Friston, 2019). By synchronously biasing the local excitability of distant circuits, these quantum-assisted events may seed the global integration and network-level reorganization observed during the psychedelic state, influencing large-scale structures such as the Default Mode Network (Muthukumaraswamy et al., 2013). This provides a mechanism for global coordination that supplements classical, transport-limited axonal signaling with a statistically correlated “excitability gate”.

In our hypothesis, Posner molecules offer a possible physical substrate linking psychedelic-induced Ca^2+^ and phosphate flux to emergent network phenomena. Psychedelics produce intense cortical activity with large calcium transients and substantial metabolic turnover, conditions that could increase the likelihood of Posner cluster formation and interaction. Mitochondria sequester calcium and phosphate during these events and may transiently generate amorphous calcium phosphate stores (Delacour et al., 2025). The connection between lithium isotopes and calcium phosphate granules suggests that spin-dependent modulation of such structures may influence neuropsychiatric states, raising the possibility that psychedelic states could also involve spin-sensitive processes in related complexes (Zadeh-Haghighi and Simon, 2021; Delacour et al., 2025).

**FIGURE 1:**
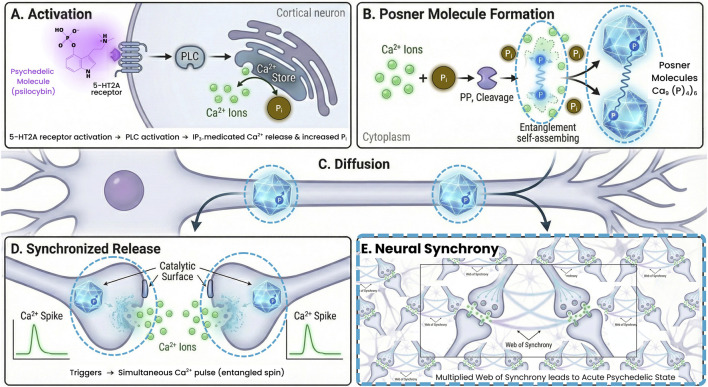
Schematic of the proposed quantum-assisted mechanism. **(A)** 5-HT2A receptor activation by a psychedelic (in this case psilocybin) leads to PLC activation and IP_3_-mediated Ca^2+^ release in a cortical neuron as well as increased metabolic phosphate (P_i_) generation. **(B)** Excess Ca^2+^ and P_i_ may form Posner molecules (Ca_9_) (PO_4_)_6_) in the neuron’s environment. Enzymatic cleavage of pyrophosphate (PP_i_) produces two entangled phosphate ions (blue glow indicates entangled spin state). These are taken up into two separate Posner molecules. **(C)** The Posner molecules diffuse to different locations (e.g., two synaptic terminals in the same neuron or in different neurons). **(D)** When Posner molecules encounter certain triggers (e.g., binding to each other or a catalytic surface), they dissolve, simultaneously releasing a pulse of Ca^2+^ ions in both locations. The entangled spin state ensures a coincident release (synchronized Ca^2+^ spikes in the two terminals). **(E)** Coincident Ca^2+^ pulse causes synchronous neurotransmitter release or neuronal firing that links the activity of the two otherwise separate neurons. Repeated occurrences could create a web of quantum-assisted synchrony across neural networks, potentially contributing to the acute psychedelic state. All Posner-related elements are hypothetical (dashed outlines); the schematic is intended to define falsifiable steps rather than assert *in vivo* existence.

The Posner hypothesis does not replace classical serotonin receptor pharmacology and neuroplasticity frameworks. It introduces an additional speculative but testable layer in which nuclear spin-dependent chemistry might influence synchrony, integration, or individual variability in psychedelic response. Classical 5-HT2A activation already explains many phenomenological and therapeutic effects through transcriptional, plasticity, and glutamatergic pathways (Vargas et al., 2023). The nuclear spin hypothesis asks whether some distinctive features of the psychedelic state might be amplified by quantum coherent molecular events. If Posner entanglement exists, it could provide a route for coordinated signaling events across distances, potentially contributing to unusual integrative experiences during psychedelic sessions. Figure 1 schematizes this proposed quantum-assisted synchrony pathway, linking receptor pharmacology to putative nuclear-spin dynamics and network effects. This remains hypothetical but is experimentally tractable and positions Posner molecules as a structured and chemically grounded platform for exploring quantum effects in the brain.

### 3.4 Quantitative constraints and feasibility bounds

To assess the physical plausibility of the proposed nuclear-spin transduction chain, we outline order-of-magnitude feasibility bounds for each stage of the proposed mechanism (Table 1). These bounds are not claims that Posner molecules exist *in vivo*; instead, they define the thermodynamic and kinetic envelope in which the mechanism could remain viable and therefore measurable. The key distinction is between an “active niche” (transient, high-flux microenvironments such as synaptic nanodomains or the mitochondrial matrix) and the basal, non-permissive environment of the resting cytosol (Fisher, 2015; Swift et al., 2018).

**TABLE 1:** Order-of-magnitude feasibility bounds for the proposed Posner-spin mechanism.

Stage	Parameter	Active niche bound (proposed)	Basal non-permissive environment	Notes on interpretation
Nucleation	Local Ca2+	0.5–1.0 mM (Transient microdomain or mitochondrial matrix peak)	∼100 nM	Reflects brief nanodomain or matrix peaks; not sustained bulk cytosolic concentration
	Local PO_4_^3-^ (activity)	1–5 mM (Transient local supersaturation/activity) (Tegmark, 2000; Barry et al., 2016)	∼1 mM	Refers to effective local activity under high metabolic flux, not bulk free phosphate
	Kinetics	Rapid nucleation under supersaturation (theoretical)	Non-nucleating	Represents thermodynamic feasibility under transient supersaturation
Persistence	Nuclear spin coherence (T_2_)	10^2^–10^3^ s (Theoretical upper bound under ideal symmetry protection^a^)	<10^–6^ s (Decoherence floor) (Tegmark, 2000)	Upper bound reflects idealized symmetry-shielded conditions; lower bound reflects environmental decoherence estimates
	Cluster lifetime	Minutes to hours (Modeled solution-phase metastability)	<1 m (Immediate dissociation)	Based on solution modeling; *in vivo* stability unknown
Transport	Effective displacement rate	∼1 μm/s (Active intracellular transport regime)	∼0.1 μm/s (Passive diffusion regime)	Order-of-magnitude transport envelope only
Dissolution	Ionic gain	∼9 Ca^2+^ per cluster (Stoichiometric release)	<1 ion (Immediate rebinding)	Directly determined by cluster composition
	Amplification	≥5-fold biochemical amplification (Conceptual estimate^b^) (Augustine et al., 2003; Neher, 1998)	<1 (Buffered)	Estimated relative to vesicle fusion sensitivity thresholds

^a^ Theoretical upper bound assuming idealized S_6_ symmetry protection and minimal environmental spin coupling.

^b^ Conceptual amplification estimate based on stoichiometric Ca2+ release relative to known vesicle fusion sensitivity; not directly measured *in vivo*.

The “active niche” represents transient, high-flux physiological regimes (e.g., synaptic nanodomains during intense Ca2+ influx or the mitochondrial matrix during peak metabolic turnover) where ionic supersaturation and structured calcium-phosphate phases may transiently become thermodynamically accessible (Fisher, 2015; Swift et al., 2018; Augustine et al., 2003; Neher, 1998). These conditions are not characteristic of the resting cytosol and would not be expected to persist beyond short time windows (Augustine et al., 2003; Neher, 1998; Vargas et al., 2023). Critically, the bounds in Table 1 assume only transient formation of Posner-like clusters under high-flux conditions; the existence, abundance, and *in vivo* lifetime of such clusters remain to be experimentally determined (Fisher, 2015; Anderson et al., 2026). Coherence estimates are reported as theoretical upper limits under ideal symmetry protection and contrasted against an expected environmental decoherence floor (Swift et al., 2018; Yunger Halpern and Crosson, 2019; Tegmark, 2000).

By formalizing these envelopes, we establish explicit falsification criteria. If direct measurements demonstrate that 5-HT2A-mediated signaling environments during psychedelic exposure remain consistently within the basal, non-permissive regime, or if calcium-phosphate clusters fail to exhibit metastability or spin persistence within these bounds, then the proposed quantum-mechanical contribution would be effectively refuted (Pikor et al., 2024; Ghibelli et al., 2010; Augustine et al., 2003).

## 4 5-HT2A receptor activation, calcium dynamics, and nuclear spin pathways

The interface between psychedelic pharmacology and Posner-related nuclear spin effects arises from calcium signaling and phosphate metabolism. Activation of 5-HT2A receptors by classical psychedelics engages the phosphoinositide cascade through G_q/11_ coupling, leading to phospholipase C (PLC) activation, PIP_2_ cleavage, and IP_3_-mediated release of Ca^2+^ from the endoplasmic reticulum. In cortical pyramidal neurons, this promotes dendritic Ca^2+^ spikes, recurrent excitatory activity, and increased glutamatergic transmission (Nichols, 2016; Kwan et al., 2022; Vargas et al., 2023). The associated metabolic demand elevates ATP turnover and glycolytic flux, producing bursts of inorganic phosphate and pyrophosphate intermediates. During intense activity, local Ca^2+^ and P_i_ concentrations may transiently exceed solubility thresholds, creating conditions under which calcium phosphate nanoclusters can nucleate, thereby creating an opportunity for nuclear spin dynamics to become relevant.

Within this context, we identify several potential interfaces between 5-HT2A signaling and Posner-based nuclear spin processes:

- High levels of Ca^2+^ and P_i_ in restricted microdomains such as synapses, dendritic spines, or intracellular stores may promote the nucleation of Posner molecules or related calcium-phosphate clusters. Psychedelic-induced Ca^2+^ surges could increase the availability of substrates required for Posner formation.
- The metabolic environment may support enzymatic cleavage of PP_i_→P_i_, a reaction proposed to generate entangled phosphate pairs (Fisher, 2015). If these phosphates enter distinct Posner molecules, the system becomes populated with clusters carrying quantum correlations.
- Posner clusters formed extracellularly could be taken up into neurons or astrocytes, while clusters formed intracellularly in mitochondrial matrix granules could be released through turnover or vesicle cycling. Psychedelics, by enhancing synaptic remodeling and vesicle dynamics, may influence the trafficking and localization of such clusters.
- Dissolution of Posner molecules provides a potential quantum readout. Shifts in pH, ionic composition, or protein binding during synaptic activity could trigger Posner disassembly. For entangled pairs this would produce correlated Ca^2+^ release events in two separate locations (Fisher, 2015).

Together, the sequence of 5-HT2A activation, Ca^2+^ and phosphate flux, Posner formation and entanglement, and Posner dissolution provides a hypothetical path through which psychedelics could couple macroscopic neural activity to nuclear spin dependent processes. In principle, correlated Ca^2+^ release events could introduce slight biases toward synchronous activity across distant sites.

Empirically, psychedelic states are marked by increased global functional connectivity and intermittent large-scale integration (Tagliazucchi et al., 2014; Carhart-Harris et al., 2014). Quantum assisted Ca^2+^ correlations, if they occur, could contribute to such phenomena by seeding occasional synchronous events between distant circuits or by subtly biasing plasticity toward patterns that reinforce these episodes. Although speculative, this mechanism offers a biochemical route for how nuclear spin dynamics might accentuate the integrative features of the psychedelic state.

Classical receptor pharmacology and intracellular signaling remain the primary drivers of psychedelic effects. Posner mediated spin dynamics, if present, would function as a background modulator that might help explain certain idiosyncrasies of experience or the variability of clinical response. The following sections consider the broader implications of this possibility and how it may complement classical frameworks in neuropharmacology.

### 4.1 Minimal circuit bridge and latency-independent coordination hypotheses

This section specifies a falsifiable circuit signature that distinguishes spin-mediated coincidence bias from purely receptor-mediated latency-constrained coordination. A central inferential challenge in linking transient molecular events to macroscopic psychedelic brain signatures lies in specifying the smallest mechanistic bridge between intracellular calcium dynamics and measurable circuit phenomena. Classical models of 5-HT2A receptor activation posit that psychedelics alter network dynamics primarily through increased excitability, reduced inhibitory tone, and changes in synaptic gain. Within such accounts, alterations in synchrony or entropy emerge through propagation along anatomical pathways and are constrained by axonal conduction delays, synaptic integration windows, and intrinsic neuronal refractory periods. Consequently, in a purely classical receptor-mediated framework, long-range coordination must scale with structural connectivity and distance-dependent latency.

We first propose a minimal biochemical-to-circuit bridge that does not rely on global depolarization. Transient, correlated calcium release events, which can arise from cluster dissolution under high metabolic flux, would produce temporally aligned increases in vesicle release probability at spatially separated synapses. Because neurotransmitter release is a nonlinear function of local calcium concentration, even modest coincident subthreshold pulses could increase the probability of near-synchronous excitatory postsynaptic potentials within narrow coincidence-detection windows. Layer V pyramidal neurons, known to act as coincidence detectors via NMDA nonlinearities and dendritic calcium spikes, would be particularly sensitive to such temporally aligned events. Repeated sparse coincidence windows would therefore increase the probability of zero-lag spike co-occurrence between distant neuronal populations without requiring enhanced axonal coupling.

This mechanism yields a distinct empirical prediction: after regressing out structural connectivity, conduction delays, and global excitability effects, there should remain a measurable residual zero-lag coincidence component between spatially separated neuronal populations. Classical receptor-mediated models predict that synchrony changes, if present, should decay with distance and remain constrained by conduction latency scaling. By contrast, the proposed coincidence mechanism predicts a distance-independent excess of near-simultaneous activation that cannot be fully explained by anatomical geometry.

To formalize this bridge, we introduce a complementary second hypothesis grounded in the latency–refractory network framework developed by Silva and colleagues (Silva, 2019). In this model, efficient signaling in structured networks depends on the relationship between signal arrival times and the residual refractory state of receiving nodes. For a neuron *v*, activation depends critically on whether incoming signals arrive within a window defined by conduction latency and intrinsic recovery dynamics. Classical coordination therefore respects geometric constraints: correlated activity must emerge through latency-aligned propagation along edges of the network.

If $R_{v}\left(t\right)$ denotes the effective refractory state of neuron v at time t , sparse, temporally aligned biochemical pulses can be modeled as a transient modulation term $g\left(t\right)$ such that

$$R_{v}\left(t\right)\to R_{v}\left(t\right)-g\left(t\right)$$ (1)

for neurons participating in a correlated event. Critically, $g\left(t\right)$ is not mediated by axonal conduction and does not depend on structural distance. Instead, it transiently lowers the effective refractory barrier across spatially separated neurons, synchronizing their coincidence-detection windows independently of white-matter pathways. Within the Silva latency–refractory framework, this constitutes a violation of purely geometry-constrained activation dynamics and provides a minimal, mechanistically explicit bridge from intracellular events to circuit-level signatures (Silva, 2019).

This latency-independent perturbation predicts a differentiated circuit signature. Specifically, measures such as spike-train cross-correlation, phase-locking value, or zero-lag coherence should reveal a residual coincidence component that persists after controlling for tract length, conduction delay, and global depolarization effects. If coordination strictly follows classical distance-decay relationships under psychedelic exposure, the proposed refractory-perturbation bridge would be falsified.

Importantly, when coupled to the nuclear spin hypothesis described earlier, this framework yields a decisive experimental test. If correlated calcium pulses arise through spin-dependent cluster dissolution, perturbations of nuclear spin coherence, such as isotope manipulation of xenon, should selectively attenuate the distance-independent residual coincidence component while leaving classical distance-dependent coordination and baseline excitability largely intact. Conversely, if nuclear spin perturbations fail to alter the residual zero-lag component, or if no such residual component is detectable after controlling for structural latency constraints, the proposed mechanism would be effectively refuted.

Together, these hypotheses provide a constrained and testable pathway from transient molecular events to measurable circuit phenomena. Rather than invoking generalized increases in synchrony or entropy, the model predicts a specific deviation from classical latency-constrained coordination, a minimal and falsifiable signature distinguishing spin-mediated coincidence detection from standard receptor-mediated accounts.

## 5 Translational implications: pharmacological and clinical significance

Although highly speculative, if the “quantum brain” Posner-based quantum mechanism hypothesis for psychedelics were validated, it would open a novel perspective on psychopharmacology, with implications for treatment variability, clinical trial design, and future drug development.

### 5.1 Inter-individual variability in treatment response

Psychedelic-assisted psychotherapy has shown promising efficacy in treatment-resistant depression, anxiety disorders, and substance use disorders, but response is significantly heterogeneous, with some patients experiencing transformative therapeutic breakthroughs, while others have milder effects or even adverse responses (Nichols et al., 2017; de Vos et al., 2021). Classical factors–dose, concurrent medications, set and setting, therapeutic alliance, and genetic variation in receptors or transporters–undoubtedly account for much of this variance. However, if nuclear spin-dependent processes play a role, subtle biological differences might also influence outcomes, suggesting additional, testable sources of variability:

- Calcium and phosphate metabolism: Differences in calcium and phosphate metabolism, which could be affected by diet, hormonal status (e.g., parathyroid hormone, vitamin D status), or genetic variants in Ca^2+^/phosphate handling, might alter the prevalence of Posner-mediated effects, modulating Posner formation and dissolution probabilities.
- Enzymes that generate or handle PP_i_ and polyphosphates: Genetic variants or disease-related changes in pyrophosphatases, alkaline phosphatases, polyphosphate kinases, or related enzymes might alter the frequency of entangled phosphate generation. For example, someone with higher enzymatic activities might form more Posner molecules during a psychedelic session, potentially leading to stronger network-level oscillations or more pronounced subjective effects.
- Interaction with lithium or other spin-active agents: Patients receiving lithium, whose isotopes show distinct behavioral effects in animal models, might exhibit systematically different responses to psychedelic therapy if nuclear-spin processes modulate both treatments (Zadeh-Haghighi and Simon, 2021; Delacour et al., 2025; Ettenberg et al., 2020).

Clinically, this suggests new lines of investigation: researchers could look for correlations between metabolic markers–phosphate levels, calcium regulatory genes, lithium exposure history–and psychedelic outcomes. Positive correlations would not prove a quantum mechanism but might hint at an underlying quantum mechanism contributing to the efficacy of psychedelic therapy in certain individuals, if more targeted experiments are conducted.

### 5.2 Novel clinical trial designs and enrichment strategies

If spin-dependent processes are relevant, several unconventional but feasible trial manipulations–isotopic manipulations or magnetic field conditions–as experimental arms in clinical trials become attractive as both tests of the hypothesis and potential levers for enrichment:

- Isotopically-modified psychedelics: Comparison of stable-isotope-substituted analogs of psilocybin, psilocin, or LSD (e.g., deuterated, ^13^C, or ^15^N-labeled). Safety, acute subjective effects, network-level neuroimaging signatures, and clinical outcomes between canonical and isotopically-modified compounds can be assessed in phase I/II studies. While isotopic changes would not be expected to dramatically alter pharmacodynamics, systemic differences in clinical response, efficacy or qualitative experience, might indicate a spin-dependent contribution. Furthermore, it could provide high-leverage tests of the hypothesis without risk to patients – ^13^C or ^2^H-labelled psychedelics are expected to be pharmacologically similar but require PK confirmation. A crossover study within subjects, comparing their experience with normal versus isotopically enriched psychedelic on separate sessions, could be illuminating if consistently one produces a deeper or faster therapeutic response. Blinding in these scenarios would be feasible–subjects would not know the difference in the drug composition.
- Magnetic field manipulations: Similarly, the environment could be varied–therapy sessions could be run under different magnetic environments (e.g., normal geomagnetic fields vs. magnetically shielded rooms vs. strong homogeneous fields with appropriate safety measures) to see if outcome measures differ. A disrupted magnetic environment would be predicted to dampen any spin-mediated contribution. Radical-pair-based mechanisms are known to be sensitive to weak magnetic fields in other systems (Hore and Mouritsen, 2016). A failure to detect any reproducible magnetic field dependence in well-controlled experiments would disfavor radical-pair-like contributions to psychedelic action.
- Adjunct lithium or other spin-active co-treatments: If this hypothesis holds true, it can be used to improve therapy. Low-dose lithium or specific lithium isotopes (^6^Li versus ^7^Li) could be explored as adjuncts in carefully monitored settings. Lithium’s therapeutic effects might synergize if it indeed enhances nuclear-spin entanglement pathways since lithium can prolong spin lifetimes or radical pairs (Zadeh-Haghighi and Simon, 2021; Ettenberg et al., 2020). Differential modulation of psychedelic efficacy or durability by lithium or lithium isotopes would strongly support common spin-dependent elements. This is presented as a mechanistic probe and would require careful safety and interaction evaluation.

From a precision-trial perspective, these manipulations could eventually serve as enrichment tools, if validated, to preferentially recruit patients whose biology is more conducive to favorable outcomes (e.g., specific mineral profiles or enzyme genotypes). At minimum, they provide powerful falsification opportunities. While these interventions are speculative, they illustrate how falsification and application can go hand-in-hand: by attempting to modulate the putative quantum aspect, we both test its existence and potentially discover a way to fine-tune therapy. Recent evidence suggests that nuclear spin modulates neurobiology via spin-sensitive calcium-phosphate nanophases, specifically mitochondrial amorphous granules (Zadeh-Haghighi and Simon, 2021; Delacour et al., 2025; Silva, 2019). We propose that Posner molecules represent a structured, coherent subset of this broader calcium-phosphate family (Fisher, 2015; Swift et al., 2018; Yunger Halpern and Crosson, 2019). This alignment suggests that while anesthetics may dampen network activity by disrupting spin-sensitive reaction channels, psychedelics—characterized by high metabolic turnover—may engage these same substrates to amplify integrative dynamics (Ettenberg et al., 2020; Silva, 2019).

This “shared substrate” model provides an immediate experimental bridge: if both phenomena tap into a common spin-sensitive biochemical layer, agents like xenon or lithium isotopes should predictably modulate 5-HT2A-mediated neural signatures (Zadeh-Haghighi and Simon, 2021; Silva, 2019). Conversely, systematically null results would demonstrate that anesthesia and psychedelic action rely on entirely distinct mechanisms, positioning this intersection as a decisive testing ground for the functional role of spin-dependent processes in the brain (Augustine et al., 2003; Anderson et al., 2026).

### 5.3 Toward “quantum-aware” CNS drug design

In the long term, if evidence accumulates that nuclear-spin processes influence CNS pharmacology, embracing a quantum perspective may inspire a new class of CNS-active compounds, with medicinal chemistry treating nuclear spin as a design parameter. Potential directions could include:

- Designing 5-HT2A agonists that optimize specific spin configurations or incorporate spin-sensitive moieties intended to maximize or probe quantum effects. A hypothetical compound could include an isotope with a long spin coherence time or one that can entangle with neural phosphorus.
- Developing agents that modulate polyphosphate metabolism or Posner-like cluster formation, potentially serving as adjuncts that tune neural synchrony in disorders of network timing (e.g., epilepsy, schizophrenia, or cognitive disorders). For example, compounds that stabilize Posner molecules or promote their uptake into neurons.
- Exploring “entangled ligand” paradigms, where pairs of molecules are engineered to become entangled via spin correlations before administration, with each member of the pair targeting different brain regions or different individuals (in a speculative “two-brain entanglement” experiment).

If quantum effects are found to play a role, it may be possible to develop biomarkers for them. EEG or MEG signatures–extremely high-frequency oscillations or anomalous phase correlations could serve as indirect indicators of quantum-coherent events. In the long run, individualized calibration like adjusting a patient’s mineral intake or using transcranial magnetic fields could be used to optimize the “quantum environment” during therapy for maximal beneficial effect. These remain very speculative notions, but they illustrate that even a small quantum contribution, if real, could be leveraged once we understand it.

While these concepts are currently far beyond clinical reality, these ideas underscore the high-reward potential: how a validated quantum contribution could usher in a quantum pharmacology paradigm where the quantum state of a drug is considered as part of its mechanism of action in addition to the chemical structure that complements receptor-targeted design. Confirming a quantum-mechanistic component in psychedelic action would expand neuropharmacology into a new dimension: it would require collaboration between neuroscientists, quantum physicists, and pharmacologists to fully exploit. Importantly, even a null result–showing that no such quantum effects occur, is valuable, as it refines the boundaries of what processes are relevant in neuropsychiatric treatment.

## 6 Experimental directions and testable predictions

**FIGURE 2:**
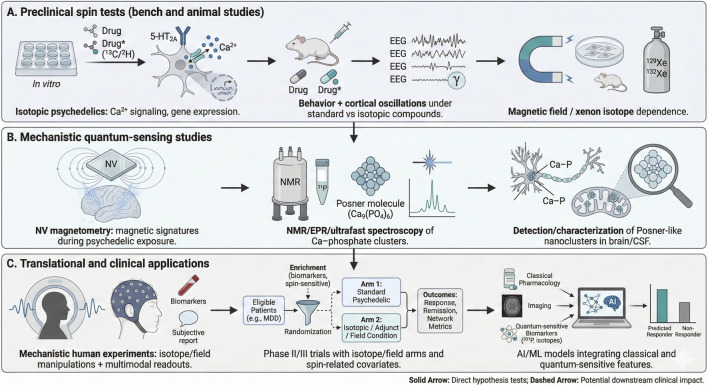
Hierarchical experimental roadmap for testing Posner-spin contributions to psychedelic action and clinical response. **(A)** Preclinical Spin Tests–Isotopic substitution (*in vitro*): Comparison of standard versus isotopically-modified (e.g., ^2^H, ^13^C, ^15^N) psychedelics to assess functional differences in 5-HT2A receptor signaling, Ca^2+^ dynamics, and transcriptomic signatures; Behavioral and Electrophysiological Models: Evaluation of behavioral proxies and (e.g., head-twitch response) and cortical oscillations in rodents to detect spin-dependent shifts *in vivo*; Magnetic and Xenon Perturbations: Testing the sensitivity of psychedelic-induced neural activity to external magnetic fields and spin-active (^129^Xe) versus spin-zero (^132^Xe) atmospheres to probe for radical pair or spin-sensitive intermediates. **(B)** Mechanistic Quantum-Sensing Studies–NV-Center Magnetometry: Utilization of diamond-based quantum sensors to detect nanoscopic magnetic transients potentially arising from synchronized Ca^2+^ currents or ^31^P nuclear spin ensembles during drug exposure; Spin-Sensitive Spectroscopy: Application of NMR, EPR, and ultrafast vibrational spectroscopy to identify Posner-like clusters and quantify ^31^P coherence times in biological environments; Structural Validation: Targeted detection and characterization of calcium-phosphate nanoclusters in neural tissue or CSF to verify the existence of proposed molecular substrates. **(C)** Translational and Clinical Applications–Mechanistic Human Studies: Assessment of the impact of isotopic or magnetic field manipulations on acute phenomenology and multimodal neuroimaging (EEG/MEG/fMRI) signatures; Precision Clinical Trial Designs: Integration of enrichment strategies into Phase II/III trials by stratifying patients based on Ca^2+^/phosphate metabolism markers or adjunct spin-active treatments (e.g., Li isotopes); AI/ML Integration: Development of predictive models that synthesize classical pharmacological data with validated quantum-sensitive biomarkers to optimize personalized treatment response. Solid arrows denote direct tests of the quantum-spin hypothesis and dashed arrows illustrate the downstream clinical utility of the program that persists even if the specific quantum mechanism is refuted.

To make the Posner-spin hypothesis scientifically actionable, we propose a decision-tree experimental program (Figure 2). These experiments are designed to distinguish between classical pharmacological activity and the proposed quantum-mechanical contributions by targeting specific physical variables, namely, nuclear spin, magnetic field, and isotopic mass, that are typically overlooked in standard neuropharmacological models.

We begin with testing substrate existence (“gating assays”), followed by probing spin sensitivity with the most discriminative perturbations (xenon isotopes, magnetic fields), and only then do we escalate to advanced quantum-sensing and translational studies. Each step is tied to a predicted observable and explicit “stop rules” (Tables 2, 3). The predictions in Table 2 are organized into an evidentiary hierarchy to ensure interpretive clarity. A well-powered null result at early gates is valuable: it efficiently constrains or refutes the specified Ca_9_(PO_4_)_6_-based mechanism.

**TABLE 2:** Primary predictions and falsification decision rules.

Prediction	Expected direction	Primary readout	Negative outcome (falsification)
Xenon Isotope Dependence	^129^Xe (*I* = 1/2) will reduce synchrony metrics or HTR magnitude relative to^132^Xe (*I* = 0) by accelerating^31^P decoherence	Rodent head-twitch response (HTR) magnitude or cortical synchrony (EEG/LFP)	No reproducible difference between^129^Xe and^132^Xe under adequately powered and magnetically controlled conditions
Magnetic Field Sensitivity	Perturbation of the ambient magnetic bath within spin-sensitive ranges will alter the kinetics of 5-HT2A signaling	Intracellular Ca^2+^ transients or downstream ERK/PLC activation in layer V neurons	Signaling and behavior remain invariant to field perturbations across ranges predicted to perturb spin coherence
Ligand Isotope Substitution	Substitution (e.g., ^2^H,^13^C) will produce signaling differences exceeding classical kinetic isotope effect expectations	Transcriptomic signatures (*c-Fos*) or durable plasticity markers	Behavioral and cellular responses remain within ranges predicted by classical mass-dependent kinetics
Structural Prevalence	Posner-like nanoclusters will be detectable in neural tissue, specifically under high metabolic flux conditions	^31^P-NMR, pulsed EPR, or CARS targeting phosphate vibrations	Failure to detect structured Ca-P clusters in brain tissue or CSF during peak 5-HT2A excitability states
Calcium Flux Amplification	Local Ca^2+^ release from dissolving clusters will produce a non-linear increase in vesicle release probability	Paired synapse coincidence probability	No measurable coincidence bias beyond classical diffusion limits or buffered baseline noise

**TABLE 3:** Isotopic control logic and matching criteria.

Manipulation	Dominant classical confounds	Minimum measurement requirements	Quantitative matching criteria (Gatekeeper)
Deuterated Ligands (^2^H vs. ^1^H)	Primary KIE: Altered metabolic t_1_/_2_ and AUC; secondary effects on receptor Kᵢ	Plasma PK (C_max_, AUC); Parent/Metabolite ratio; *In vitro* Kᵢ and E_max_	Dose-adjustment required to achieve plasma AUC within **±15%**; Kᵢ variance **<10%**
Heavy Isotopes (^13^C,^15^N)	Minimal; potential subtle shifts in vibrational modes or solvation energy	Plasma PK profile; HPLC-MS metabolite quantification	Plasma exposure metrics (AUC/C_max_) must meet standard bioequivalence (**80%–125%**)
Xenon Isotopes (^129^Xe vs. ^132^Xe)	Physical Confounds: Negligible; minor gas diffusion/viscosity differences	Inspired gas fraction (Fᵢ); Blood-gas partial pressure (P_xe_)	Equalized molar fractions; identical P_xe_ sustained throughout exposure window
Phosphorus Isotope (Theoretical^32^P)	N/A: Used primarily as a structural tracer or in *in vitro* cluster modeling	Scintillation counting or NMR signal integration. (*In vitro* validation only)	Total cluster-embedded phosphorus concentration must be normalized to within **±5%**

Bold values indicate thresholds that must be met before any observed isotope-dependent difference is interpreted as evidence for a potential nuclear-spin contribution rather than a classical pharmacokinetic or physicochemical confound.

To prevent classical Kinetic Isotope Effects (KIE) and pharmacokinetic (PK) variance from confounding the interpretation of nuclear-spin-dependent results, we establish a strict control logic for all isotopic experiments (Table 3). A difference in signaling or behavior is only attributed to nuclear spin if the exposure and target-engagement criteria are met within the same study.

### 6.1 Substrate validation (“gating assays”) in the active niche

To address the ontological status of the Posner molecule in the brain, we elevate substrate validation to a primary requirement to ensure the research program is grounded in structural reality. We treat substrate detection in the active niche as a gating requirement. A consistent null result in both gating assays would constitute a fundamental refutation of the specific Ca_9_(PO_4_)_6_ substrate proposed in this work, regardless of any downstream behavioral or electrophysiological observations.

#### 6.1.1 High-field ^31^P NMR spectroscopy (structural signature Screen)

- Protocol: High-resolution ^31^P NMR on cortical slices or purified mitochondrial fractions after acute 5-HT2A agonist exposure (with matched controls).
- Positive Detection: Detection of a specific resonance peak or characteristic muliplet structure at the chemical shift predicted for the S_6_-symmetric Posner environment, distinguishable from bulk inorganic phosphate and phospholipid signals.
- Negative (Gating Falsification): No evidence of any structured ^31^P signal corresponding to inorganic nanoclusters in high-flux samples, indicating that phosphate remains in a purely disordered amorphous or soluble state.

#### 6.1.2 Cryo-electron microscopy (Cryo-EM) with elemental mapping (direct nanophase detection)

- Protocol: Cryo-EM of rapidly frozen layer V pyramidal tissue to detect ∼1 nm clusters (Anderson et al., 2026).
- Positive Detection: Detection of roughly spherical, 0.9–1.0 nm nanoclusters within the mitochondrial matrix or synaptic nanodomains with Energy-Dispersive X-ray (EDX) spectroscopy confirming a localized Ca:P ratio consistent with 9:6 stoichiometry.
- Negative (Gating Falsification): Failure to identify any structured calcium-phosphate nanophases under conditions that maximize Ca^2+^ and metabolic flux, arguing that the substrate lacks the necessary stability to serve as a quantum memory element.

### 6.2 Highest-leverage discriminative tests of spin sensitivity

While we maintain a comprehensive experimental roadmap to fully characterize potential quantum-biochemical interfaces, we explicitly prioritize a subset of near-term, discriminative tests designed to provide high-leverage falsification opportunities (Figure 2). The most immediate of these for the Posner-spin hypothesis involve perturbations of the nuclear spin environment and the ambient magnetic bath (Fisher, 2015).

#### 6.2.1 Xenon isotope crossover (spin vs. spin-zero)

We propose crossover experiments that leverage the nuclear spin differences across chemically indistinguishable ^129^Xe (*I* = 1/2) and ^132^Xe (*I* = 0) isotopes (Li et al., 2018). We exploit this difference by predicting that if ^31^P spin-sensitive steps contribute, ^129^Xe in neural tissue may interact with the ^31^P nuclear spins within Posner molecules through a weak but measurable dipole-dipole coupling that accelerates the loss of spin coherence our model requires relative to ^132^Xe under matched conditions. Any reproducible difference in 5-HT2A-mediated signaling or behavior between preparations exposed to these two otherwise identical isotopes would therefore constitute evidence that the signaling cascade is sensitive to nuclear spin state, a result that is very difficult to account for through classical biochemistry alone. A failure to observe reproducible differences in 5-HT2A-mediated signaling or behavior under ^129^Xe compared to ^132^Xe would constitute a strong falsification of the Posner-based substrate.

To ensure that any observed differences between the xenon isotopes are uniquely attributable to nuclear spin coupling rather than classical sedative or network-suppressant effects, we implement a multi-tiered control and monitoring protocol.

Arousal and Sedation Monitoring: To exclude global network suppression as a confound, all isotope crossover experiments will include real-time electrophysiological monitoring. We will track the power spectra of EEG/LFP signals, specifically monitoring the Delta (0.5–4 Hz) and Gamma (30–80 Hz) bands. Classical xenon-induced sedation is marked by a characteristic increase in Delta power and a global suppression of high-frequency activity; a spin-dependent psychedelic effect, conversely, is predicted to manifest as a shift in 5-HT2A-mediated desynchronization without a corresponding shift in the “arousal floor” between isotopes (Li et al., 2018).

Behavioral Controls: In rodent models, we will employ concurrent motor activity monitoring (e.g., open-field distance) to ensure that HTR measurements are not confounded by motor impairment. If ^129^Xe reduces the magnitude of the 5-HT2A-mediated HTR while both isotopes produce identical reductions in total locomotor activity, the result would provide strong evidence for a spin-specific interaction with the psychedelic signaling chain rather than a generalized anesthetic effect.

Predicted Direction and Plausible Magnitude: Based on the hypothesis that ^129^Xe (*I* = 1/2) disrupts ^31^P coherence through dipole-dipole coupling, the predicted direction of the effect is an attenuation of the psychedelic-induced state. Specifically, ^129^Xe is expected to weaken the quantum-assisted synchronization of Ca^2+^ transients, thereby reducing the intensity of behavioral and neural readouts compared to the magnetically silent ^132^Xe (*I* = 0). Drawing from previously reported xenon isotope effects in anesthesia, we anticipate that any spin-dependent modulation of psychedelic action, if present, should be detectable at magnitudes comparable to known xenon isotope differentials in neural potency, though the precise scaling between anesthetic and psychedelic regimes remains an open empirical question (Li et al., 2018).

#### 6.2.2 Magnetic field sensitivity

We prioritize evaluation of 5-HT2A signaling under shielded vs. ambient vs. high-field conditions. Given that spin-sensitive mechanisms such as radical-pair chemistry are field-dependent, a lack of magnetic sensitivity across relevant ranges would strongly undermine the proposed quantum mechanism (Li et al., 2018). Field ranges should be selected based on radical-pair/nuclear-spin relevant regimes (Hore and Mouritsen, 2016; Zadeh-Haghighi and Simon, 2022).

#### 6.2.3 Ligand isotope substitution as a “spin-awareness” probe

Ligand isotope substitution (e.g., ^2^H, ^13^C, ^15^N) in psychedelics serves as a near-term probe for the “spin-awareness” of the signaling cascade itself. While the ligand is not the primary quantum substrate, its nuclear spin configuration can alter the local hyperfine environment of signaling intermediates. *In vitro* studies should assess whether stable isotopically-modified psychedelics (^2^H, ^13^C, ^15^N) differ from standard formulations in 5-HT2A signaling outputs, including Ca^2+^ imaging and ERK/PLC activation, and whether substitution alters downstream Ca^2+^ dynamics or gene expression beyond modest kinetic differences. *In vivo*, animal models can assess behavioral effects such as the head-twitch response in mice and cortical electrophysiology under standard versus isotopically-modified compounds, with crossover designs used to maximize sensitivity to subtle effects. For human pilot studies, small double-blind crossover trials can compare acute phenomenology, EEG/MEG/fMRI signatures, and clinical outcomes between standard and isotopically-labeled formulations with matched pharmacokinetics. Interpretation of these experiments would require the PK bioequivalence gate (Table 3). A null result here represents a localized falsification, indicating that the signaling chain does not maintain spin-sensitivity, even if the ^31^P substrate remains theoretically conceivable. However, robust or consistent differences across these tiers would strongly motivate deeper mechanistic investigation.

For ligand-based studies (e.g., deuterated psilocybin), the dominant confound is the suppression of CYP450-mediated metabolism, which typically extends the drug’s half-life and increases systemic exposure. We propose a “Bioequivalence Gate” protocol: if the deuterated compound produces an AUC significantly outside the 80%–125% range of the standard compound, the dose must be titrated downward until plasma equivalence is reached. Only after this classical exposure is matched can a divergence in qualitative neural signatures (e.g., EEG phase-coherence) or intensity of treatment response be analyzed as a putative spin effect. The isotope effect must exceed the magnitude predicted by classical kinetic isotope theory to be considered evidence for spin-dependent contributions.

By leading with these discriminative tests, we ensure that the subsequent, more complex paradigms for cluster characterization—such as NV-center magnetometry or ultrafast spectroscopy—remain grounded in a baseline confirmation of spin-dependent signaling (Vargas et al., 2023; Zhang et al., 2023).

### 6.3 Medium-term experiments with emerging quantum-sensing technology

#### 6.3.1 NV-center diamond magnetometry

Nitrogen-vacancy (NV) centers in diamond are quantum sensors that can detect extremely small magnetic fields, including those from single neurons and potentially from small clusters of nuclear spins (Zhang et al., 2023). Recent work has demonstrated optical detection of single-neuron action potentials and optimization of NV magnetometry for neurography (Barry et al., 2016). One application is to use NV magnetometry to search for unusual magnetic noise or oscillations in brain tissue during psychedelic exposure. For example, placing an NV-diamond probe adjacent to hippocampal or cortical slices perfused with psychedelics to look for anomalous magnetic noise or oscillatory signatures that do not correlate with classical electrical activity. If entangled Posner molecules are releasing Ca^2+^ in bursts, they might produce tiny magnetic transients from synchronized ionic currents that could be picked up. Another approach is to detect magnetization or coherence signatures in chemically prepared Posner-like clusters possibly generated from brain extract. While challenging, these experiments could provide direct or indirect evidence of non-classical spin dynamics associated with psychedelic exposures.

#### 6.3.2 Ultrafast or spin-resonance spectroscopy

Two-dimensional infrared spectroscopy or electron/magnetic resonance can be used to probe potential coherence in biological phosphate compounds. For example, ultrafast laser pulses could be used on a sample of neurons or glia treated with a psychedelic to see if any long-lived oscillatory signals emerge in the terahertz or gigahertz range–signatures of coherent spin or vibrational states. Additionally, pulsed EPR/electron-nuclear double resonance (ENDOR) can be used on brain samples to check for radical-pair intermediates during psychedelic metabolism or signaling. If 5-HT2A leads to any radical formation (some G_q_ pathways produce ROS), one could see if their spin dynamics are altered by isotopic substitution. Another idea is coherent anti-Stokes Raman spectroscopy (CARS) targeting phosphate vibrations to detect if Posner clusters form. Though these experiments are complex, they could directly reveal if any coherent quantum processes accompany the biochemical actions of psychedelics.

### 6.4 Long-term experimental vision

The following concepts are far beyond current feasibility, but serve to illustrate how, if even modest evidence accumulates, more ambitious tests might eventually be conceived.

#### 6.4.1 Entangled ligand pairs

In the future, one could imagine engineering pairs of a psychedelic or related compound whose nuclear spins are entangled prior to administration to an animal or brain region and looking for any correlations in neural or behavioral responses that defy ordinary explanation. This would be a definitive proof-of-principle for quantum brain effects. Alternatively, a simpler within-brain approach would be to use entangled photons to trigger simultaneous uncaging of molecules in distant brain regions, testing whether entanglement versus classically coincident release yields different neurophysiological outcomes. While currently out of reach, requiring coordination between quantum optics and neurobiology, such “Bell-type” experiments would offer conceptually decisive tests for quantum contributions to brain function.

#### 6.4.2 Quantum-enhanced neuroimaging

If quantum processes are found to be relevant, new neuroimaging modalities could be designed to help visualize them. This could include MRI sequences sensitive to entangled spin states or new contrast agents that highlight areas of prolonged spin coherence (e.g., hyperpolarized ^31^P MRI sequences optimized for neural phosphate). Another approach would be quantum-enhanced MEG/EEG, where entangled sensor arrays improve the detection of weak, synchronous signals. These advancements could lead to the ability to map when and where in the brain unusual quantum-coherent states, if any, occur–potentially revealing a transient “burst of coherence” in association with peak psychedelic experience. Though highly speculative, pursuing these ideas pushes technology forward, and even if the hypothesis is disproven, may yield new tools for neuroscience.

### 6.5 Testability and falsifiability

A good scientific hypothesis must be falsifiable–thus a crucial aspect of this proposal is that it can be clearly unsupported or refuted. Here we outline clear criteria by which the proposed quantum Posner hypothesis for psychedelics would be falsified:

- Null isotope effects: If carefully controlled, *in vitro*, animal, and human studies reveal no reproducible differences between standard and isotopically modified psychedelics beyond what conventional chemistry predicts, nuclear-spin contributions to mechanism would be strongly undermined. For example, if ^2^H-LSD and ^1^H-LSD produce identical receptor signaling, behavioral outcomes, and therapeutic results within experimental error, then nuclear spin is likely irrelevant to the drug’s action–since ^2^H has spin one and ^1^H has spin ½ and similarly with replacing phosphorus in a psychedelic. While the changes could be too subtle to detect clinically, with large sample sizes even subtle effects should become apparent. A consistent null result on isotopic experiments would refute the idea that quantum spin dynamics contribute meaningfully.
- No magnetic field dependence: If psychedelic-induced signaling and behavioral effects are insensitive to magnetic field manipulations across relevant ranges, spin-sensitive radical-pair or Posner mechanisms become unlikely. For example, if psychedelics are administered to cell cultures or animals in various magnetic conditions (shielded versus ambient versus high field) and no differences in signaling or behavior are observed, it implies no magnetic-spin-sensitive step in the mechanism. Given that known quantum biological mechanisms are typically field-sensitive, a lack of any field effect suggests an absence of coherent spin phenomena (Zadeh-Haghighi and Simon, 2021). Of course, however, the field strengths and durations used must be sufficient to perturb spin dynamics as predicted by theory.
- Absence of Posner clusters in neural contexts: If sensitive structural and spectroscopic methods fail to identify Posner-like calcium phosphate clusters in brain tissue or CSF, the specific Posner mechanism proposed here would be untenable. This could be investigated by ultrasensitive detection methods (e.g., neutron scattering, specialized staining, or spectroscopy in brain slices). If none are found even during high-activity states, the Posner hypothesis is likely incorrect or irrelevant.
- Rapid decoherence of ^31^P: If theoretical modeling and experimental data converge on decoherence timescales that are far too short (e.g., nanoseconds or microseconds) for ^31^P spin states in the brain’s temperature and electromagnetic noise environments, then they could not realistically modulate millisecond-scale neuronal events. Some theoretical studies might conclude that the brain’s warm and wet conditions are too hostile for the required coherence time–if compelling and experimental attempts to measure Posner spin lifetimes find them to be vanishingly short, the idea of functional quantum coherence influencing neural signaling becomes unstable.
- Satisfactory classical explanations: If all the phenomena initially motivating the hypothesis–such as lithium isotope effects or unusual psychedelic network dynamics–are sufficiently explained by classical mechanisms, invoking quantum processes would render the quantum hypothesis unnecessary. For example, if ^6^Li versus ^7^Li differences were fully explained by a subtle difference in ion channel kinetics, a non-quantum effect, or if psychedelic-induced synchrony were fully explained by classical neuromodulatory feedback loops, then adding a quantum story would be extraneous. Science tends toward parsimony–without clear anomalies requiring a quantum explanation, the hypothesis would not hold merit.

Conversely, certain findings would provide incremental support, though not outright “proof”, that would warrant further investigation. These would include:

- Robust isotope-dependent differences in psychedelic effects (pharmacological or clinical).
- Consistent magnetic field effects or spin-polarization on 5-HT2A signaling or psychedelic-induced neural activity.
- Detection of Posner molecules *in vitro*, particularly if prevalence changes with neural activity or drug state.
- Observation of anomalous synchronous neural firing or Ca^2+^ transients that cannot be explained by classical connectivity.

In weighing evidence, the bar for accepting a quantum brain hypothesis is, and should be, extremely high. Extraordinary claims require extraordinary evidence. This hypothesis will likely be discarded if these experiments yield null results–still a beneficial outcome for science as it sharpens our understanding by ruling out possibilities. On the other hand, even small positive indications would open fascinating new questions and demand replication and deeper investigation.

## 7 Discussion

We have presented a speculative but experimentally-grounded hypothesis linking psychedelic pharmacology to quantum-mechanical processes involving nuclear spins and Posner molecules in the brain. This proposal draws on three lines of work: the well characterized 5-HT2A mediated Ca^2+^ signaling cascades that drive neuroplasticity and large-scale network dynamics (Nichols, 2016; Kwan et al., 2022); evidence that nuclear spin and isotope dependent effects can influence neurobiological processes (Zadeh-Haghighi and Simon, 2021; Delacour et al., 2025; Ettenberg et al., 2020); and theoretical and computational studies suggesting that Posner molecules may preserve ^31^P nuclear spin coherence under physiologically relevant conditions (Fisher, 2015; Swift et al., 2018; Yunger Halpern and Crosson, 2019). Integrating these strands, we proposed that psychedelic induced Ca^2+^ flux and phosphate turnover could increase the formation of Posner-like clusters and thereby create a biochemical setting in which nuclear spin dynamics may bias neural synchrony or clinical response. Classical receptor pharmacology and systems neuroscience explain much of the psychedelic state, and the present framework is intended to complement rather than supplant these models.

Our approach balances speculation with testability. The hypothesis is high risk because it may ultimately prove that no quantum mechanical contributions are required to explain psychedelic action. However, it is also high reward because any verification of spin-dependent processes influencing neuronal signaling would alter core assumptions in neuropharmacology and cognitive neuroscience. Even negative findings will have value, since attempts to test these ideas will yield insights into calcium handling, phosphate metabolism, and new methods for probing fast molecular events in neural tissue.

A conservative view is that the complexity of brain function arises entirely from classical interactions among ions, receptors, and circuits, and that any quantum effects decohere too rapidly to influence neural dynamics. The burden of proof therefore lies with any quantum brain hypothesis. We consider this possibility because several observations hint that spin-dependent chemistry might be biologically relevant. Lithium isotopes differ in behavioral and biochemical effects, and rapid synchronization phenomena in neural networks raise questions about potential microphysical influences (Zadeh-Haghighi and Simon, 2021; Delacour et al., 2025; Ettenberg et al., 2020). Psychedelics produce extreme states of excitability and substantial Ca^2+^ transients and metabolic flux, precisely the regime in which otherwise negligible quantum effects, if present at all, would be most visible. Even a subtle quantum bias affecting the timing of Ca^2+^ events at critical nodes could in principle influence population level dynamics.

There are, however, significant limitations. The existence, abundance, and lifetime of Posner molecules *in vivo* remain unproven. Although Ca_9_(PO_4_)_6_ clusters have been modeled and observed in simulated body fluids and amorphous calcium phosphate granules are well documented in mitochondria, direct detection of Posner molecules in neural tissue has not been achieved (Swift et al., 2018; Posner and Betts, 2002; Pivovarova and Andrews, 2010). The mechanism of entanglement generation through pyrophosphate hydrolysis and of quantum readout through Posner binding and dissolution are also theoretical. These steps must be demonstrated in simplified chemical systems and then in cells before they can be considered plausible *in vivo*. Decoherence time estimates remain uncertain, and even if Posner-mediated entanglement exists, its influence may be negligible relative to the noise and redundancy of cortical circuits. Other quantum substrates, such as radical pairs involving electron spins, may also be relevant. The radical-pair mechanism is a leading model for magnetoreception, and psychedelic-induced metabolic stress could, in principle, generate transient radicals whose spin dynamics modulate signaling pathways (Hore and Mouritsen, 2016; Zadeh-Haghighi and Simon, 2021). Exploring such alternatives represents a parallel research direction.

From a pharmacologic perspective and clinical perspective, this hypothesis is compatible with established models of psychedelic action. Clinicians do not require quantum mechanics to use psychedelic therapy effectively. The 5-HT2A-centered neuroplasticity framework already accounts for many therapeutic effects (Nichols et al., 2017; de Vos et al., 2021). If nuclear spin effects contribute even marginally, they may help explain response heterogeneity arising from differences in Ca^2+^ and phosphate homeostasis, mitochondrial calcium phosphate handling, or prior exposure to spin active agents (Delacour et al., 2025). They could also motivate experimental manipulations such as isotopic substitutions or controlled magnetic environments, which would serve both mechanistic and potential enrichment purposes. Should any quantum contributions be validated, they may eventually influence CNS drug design by introducing nuclear spin as a parameter alongside receptor affinity and pharmacokinetics.

The broader scientific value of this hypothesis lies in its falsifiability and its interdisciplinary potential. Testing it will require collaboration between quantum physicists, chemists, and neuroscientists, and will likely drive the development of new measurement tools and theoretical models. The hypothesis generates specific predictions concerning isotope effects, magnetic field sensitivity, the presence and behavior of Posner clusters, and the coherence properties of ^31^P nuclei (Fisher, 2015). Systematic failures to observe these phenomena would be decisive grounds for dismissal. Positive results, even if modest, would prompt substantial revision of foundational assumptions in neuropharmacology.

We view this work as an invitation for rigorous scrutiny. The hypothesis reframes psychedelic pharmacology not simply as receptor binding and network modulation, but as a possible test bed into evaluating whether quantum mechanical processes play any functional role in the brain. By articulating a testable framework, we hope to accelerate the empirical inquiry that will either define the limits of quantum influence in neural systems or reveal new principles governing how powerful interventions like psychedelics reshape neural circuits and subjective experience.

## 8 Conclusion

We have reformulated a broad, speculative idea–psychedelics and the quantum brain–into a series of concrete, falsifiable hypotheses centered on Posner molecules and nuclear-spin dynamics. In this framework, classic psychedelics, via intense 5-HT2A-mediated Ca^2+^ signaling and metabolic flux, could hypothetically facilitate the formation and entanglement of Posner clusters, whose dissolution might introduce subtle, non-classical correlations in neuronal activity. This hypothesis bridges translational pharmacology and quantum biology, suggesting that phenomena like inter-individual variability in psychedelic therapy and the extraordinary cognitive effects of these drugs might find partial explanation in the quantum domain. As a high-risk, high-reward hypothesis, we expect the default expectation to assume that classical mechanisms will ultimately suffice. Nonetheless, the relatively small number of focused experiments required to test the key predictions make this a scientifically worthwhile pursuit. Even so, disproving the necessity of quantum effects in brain function is a worthwhile result. On the other hand, if some facet of this hypothesis is affirmed, it could usher a paradigm shift for both neuroscience and quantum science.

## Data Availability

The original contributions presented in the study are included in the article/supplementary material, further inquiries can be directed to the corresponding author.
